# Two new species of *Geejayessia* (Hypocreales) from Asia as evidenced by morphology and multi-gene analyses

**DOI:** 10.3897/mycokeys.42.27664

**Published:** 2018-11-06

**Authors:** Zhao-Qing Zeng, Wen-Ying Zhuang

**Affiliations:** 1 State Key Laboratory of Mycology, Institute of Microbiology, Chinese Academy of Sciences, Beijing 100101, PR China Institute of Microbiology, Chinese Academy of Sciences Beijing China

**Keywords:** Cosmospora-like fungi, Nectriaceae, Systematic, Taxonomy

## Abstract

Two new species of *Geejayessia* are introduced, based on materials collected from central China. *Geejayessiaclavata***sp. nov.** is characterised by gregarious, red brownish to dark red, oval-subglobose to globose perithecia that are formed on a basal stroma; (4–7-)8-spored cylindrical asci; ellipsoidal or rarely broadly ellipsoidal, uniseptate, smooth or finely verruculose ascospores; clavate, aseptate microconidia and absence of macroconidia. *Geejayessiasinica***sp. nov.** is characterised by red to bright red, pyriform, subglobose to globose, perithecia on a basal stroma, collapsing laterally when dry; subcylindrical to clavate asci with a rounded apex; ellipsoidal, uniseptate ascospores; and falcate, multiseptate macroconidia with an arcuate tip. Morphological distinctions of the new species from the related fungi are discussed. This is the first report of *Geejayessia* from Asia.

## Introduction

Some fusarium-like species having gregarious, multicoloured, broadly ampulliform short-necked or broadly ellipsoidal perithecia were previously placed in *Cosmospora* Rabenh. and *Nectria* (Fr.) Fr. (Booth 1959; Samuels and Rogerson 1984; Nirenberg and Samuels 2000) until the genus *Geejayessia* Schroers, Gräfenhan & Seifert, typified by *G.cicatricum* (Berk.) Schroers, was introduced (Schroers et al. 2011). The genus is characterised by prosenchymatous stromata erumpent through substrates, caespitose, broadly pyriform, pale orange, brownish to reddish-orange, bright red to black perithecia, reacting to potassium hydroxide (KOH) and lactic acid (LA); cylindrical or clavate asci with eight ascospores; broadly ellipsoidal to ellipsoidal ascospores that are uniseptate, slightly constricted at the septum, hyaline or pale brown to yellowish-brown, smooth or verruculose at maturity; and multiseptate, slightly curved macroconidia with conspicuous pedicellate foot cell (Schroers et al. 2011; Lombard et al. 2015). Members of *Geejayessia* exhibit host specificity and mainly occur on *Buxus* spp., *Celtisoccidentalis* and *Staphyleatrifolia* and were reported only from Europe, North America and Oceania (Samuels and Rogerson 1984; Nirenberg and Samuels 2000; Schroers et al. 2011).

In our examinations of nectriaceous collections from central China, two cosmospora-like fungi were encountered. Judging by perithecial gross morphology, anatomic structures and culture characteristics, they represented two previously undescribed species of *Geejayessia*. Their taxonomic placements were confirmed by multigene phylogenetic analyses. Distinctions between the new species and their closely related fungi are discussed.

## Materials and methods

### Sampling and morphological studies

Specimens were collected from Shennongjia National Nature Reserve and Longyuwan National Forest Park and were deposited in the Herbarium Mycologicum Academiae Sinicae (**HMAS**). Methods used by Luo and Zhuang (2010) and Schroers et al. (2011) were generally followed for morphological observations. The test for colour changes of the perithecial wall was made with 3% KOH and 100% LA. To observe internal and microscopic characteristics of the perithecial wall, longitudinal sections through ascomata were made with a freezing microtome (YD-1508-III, Jinhua, China) at a thickness of 6–8 μm. Microscopic examinations and measurements were taken from longitudinal sections and squash mounts in lactophenol cotton blue solution using an Olympus BH-2 microscope (Tokyo, Japan). Photographs were taken with a Leica DFC450 digital camera (Wetzlar, Germany) attached to a Leica M125 stereomicroscope (Milton Keynes, UK) for gross morphology and a Zeiss AxioCam MRc 5 digital camera (Jena, Germany) attached to a Zeiss Axio Imager A2 microscope (Göttingen, Germany) for anatomical structures. Measurements of individual structures were based on 30 units, except when otherwise noted. Cultures were obtained by single ascospore isolation from fresh ascomata. To determine colony features, isolates were grown on cornmeal dextrose agar [CMD, 4% (w/v) cornmeal + 2% (w/v) dextrose + 2% (w/v) agar], potato dextrose agar [PDA, 20% (w/v) potato + 2% (w/v) dextrose + 2% (w/v) agar] and synthetic nutrient-poor agar (SNA; Nirenberg 1976) in 90 mm plastic dishes at 25 °C for 7 d. For the observation of conidiophores, macroconidia and microconidia, cultures were grown on SNA at 25 °C with alternating periods of light/darkness (12 h/12 h). Colony growth rates were measured after 7 d.

### DNA extraction, PCR amplification and sequencing

The genomic DNA was extracted from fresh mycelium following the methods of Wang and Zhuang (2004). Three primer pairs, acl1-230up/acl1-1220low (Gräfenhan et al. 2011), ITS5/ITS4 (White et al. 1990) and fRPB2-5F/fRPB2-7cR (Liu et al. 1999) were used to amplify the sequences or partial sequences of the larger subunit of the ATP citrate lyase (ACL1), the internal transcribed spacers with the 5.8S nuclear ribosomal DNA (ITS) and the second largest subunit of the RNA polymerase II (RPB2), respectively. PCR reactions were performed on an ABI 2720 Thermal Cycler (Applied Biosciences, Foster City, California, USA), based on the procedures detailed in Gräfenhan et al. (2011), White et al. (1990) and Liu et al. (1999). DNA sequencing was carried out in both directions on an ABI 3730XL DNA Sequencer (Applied Biosciences).

### Sequence alignment and phylogenetic analyses

Newly generated sequences and those retrieved from GenBank are listed in Table 1. *Nalanthamalapsidii* (Sawada & Kuros.) Schroers & M.J. Wingf. and *Thyronectriaconcentrica* (Mont. & Fr.) Voglmayr & Jaklitsch were used as outgroup taxa. Sequences were assembled, aligned and the primer sequences were trimmed with BioEdit 7.0.5 (Hall 1999) and converted to NEXUS files by ClustalX 1.8 (Thompson et al. 1997). The partition homogeneity test of ACL1, ITS and RPB2 regions was performed with PAUP 4.0b10 (Swofford 2002). To confirm the phylogenetic positions of the new species, sequences of ACL1, ITS and RPB2 were combined and analysed with Bayesian Inference (BI) and Maximum Parsimony (MP) analyses. The MP analysis was performed with PAUP 4.0b10 (Swofford 2002) using 1000 replicates of heuristic search with random addition of sequences and subsequent TBR (tree bisection and reconnection) branch swapping. Topological confidence of resulted trees was tested by maximum parsimony bootstrap proportion (MPBP) with 1000 replications, each with 10 replicates of random addition of taxa. The BI analysis was conducted by MrBayes 3.1.2 (Ronquist and Huelsenbeck 2003) using a Markov chain Monte Carlo algorithm. Nucleotide substitution models were determined by MrModeltest 2.3 (Nylander 2004). GTR+I+G was shown to be the best-fit model for the combined sequences in the BI analysis. Four Markov chains were run simultaneously for 1,000,000 generations with the trees sampled every 100 generations. A 50% majority rule consensus tree was computed after excluding the first 2500 trees as ‘burn-in’. Bayesian inference posterior probability (BIPP) was determined from the remaining trees. Trees were examined in TreeView 1.6.6 (Page 1996). BIPP greater than 90% and MPBP greater than 50% are shown at the nodes.

**Table 1.** List of species, herbarium/strain numbers and GenBank accession numbers of materials used in this study.

Species	Herbarium/strain numbers	GenBank accession numbers		
		acl1	ITS	*rpb*2
*Albonectriaalbosuccinea* (Pat.) Rossman & Samuels	BBA 64502	HQ897837	HQ897788	HQ897699
*A.rigidiuscula* (Berk. & Broome) Rossman & Samuels	CBS 122570	HQ897896	HQ897815	HQ897760
*Cyanonectriabuxi* (Fuckel) Schroers, Gräfenhan & Seifert	CBS 125554	HM626629	HM626660	HM626688
*C.cyanostoma* (Sacc. & Flageolet) Samuels & P. Chaverri	CBS 101734	HQ897895	FJ474076	HQ897759
*Dialonectriaepisphaeria* (Tode) Cooke	CBS 125494	HQ897892	HQ897811	HQ897756
*D.ullevolea* Seifert & Gräfenhan	CBS 125493	HQ897918	KM231821	HQ897782
*Fusariumsambucinum* Fuckel	CBS 14695	KM231015	KM231813	KM232381
*F.sublunatum* Reinking	BBA 62431	HM897916	HQ897830	HQ897780
*Fusicollaacetilerea* (Tubaki, C. Booth & T. Harada) Gräfenhan & Seifert	BBA 63789	HQ897839	HQ897790	HQ897701
*F.matuoi* (Hosoya & Tubaki) Gräfenhan & Seifert	CBS 58178	HQ897858	KM231822	HQ897720
*Geejayessiaatrofusca* (Schwein.) Schroers & Gräfenhan	CBS 125505	HM626628	HM626659	HM626682
*G.celtidicola* Gräfenhan & Schroers	CBS 125502	HM626625	HM626657	HM626685
*G.cicatricum* (Berk.) Schroers	CBS 125552	HQ728171	HQ728145	HQ728153
*G.desmazieri* (De Not. & Becc.) Schroers	CBS 125507	HM626633	HM626651	HM626675
*G.clavata* Z.Q. Zeng & W.Y. Zhuang	HMAS 248725	**KY873305** ^a^	**KY873307**	**KY873309**
*G.sinica* Z.Q. Zeng & W.Y. Zhuang	HMA S248726	**KY873306**	**KY873308**	**KY873310**
*G.zealandica* (Cooke) Schroers	CBS 11193	HM626626	HM626658	HM626684
*Macroconialeptosphaeriae* (Niessl) Gräfenhan & Schroers	CBS 100001	HQ897891	HQ897810	HQ897755
*M.papilionacearum* (Seaver) Gräfenhan & Seifert	CBS 125495	HQ897912	HQ897826	HQ897776
*Microceracoccophila* Desm.	CBS 31034	HQ897843	HQ897794	HQ897705
*M.diploa* (Berk. & M.A. Curtis) Gräfenhan & Seifert	BBA 62173	HQ897899	HQ897817	HQ897763
*Nalanthamalapsidii* (Sawada & Kuros.) Schroers & M.J. Wingf.	CBS 116952	KM231073	AY864836	KM232401
*Neocosmosporaramosa* (Bat. & H. Maia) L. Lombard & Crous	CBS 50963	KM231004	KM231802	KM232369
*N.vasinfecta* E.F. Sm.	CBS 32554	KM231005	KM231803	KM232370
*Stylonectriaapplanata* Höhn.	CBS 125489	HQ897875	HQ897805	HQ897739
*S.purtonii* (Grev.) Gräfenhan	DAOM 235818	HQ897919	HQ897831	HQ897783
*Thyronectriaconcentrica* (Mont. & Fr.) Voglmayr & Jaklitsch	CBS 47469	KM231080	KM231835	KM232408

^a^ Numbers in bold indicate the newly provided sequences.

## Results

### Sequence comparison and phylogenetic inference

**Figure 1.**
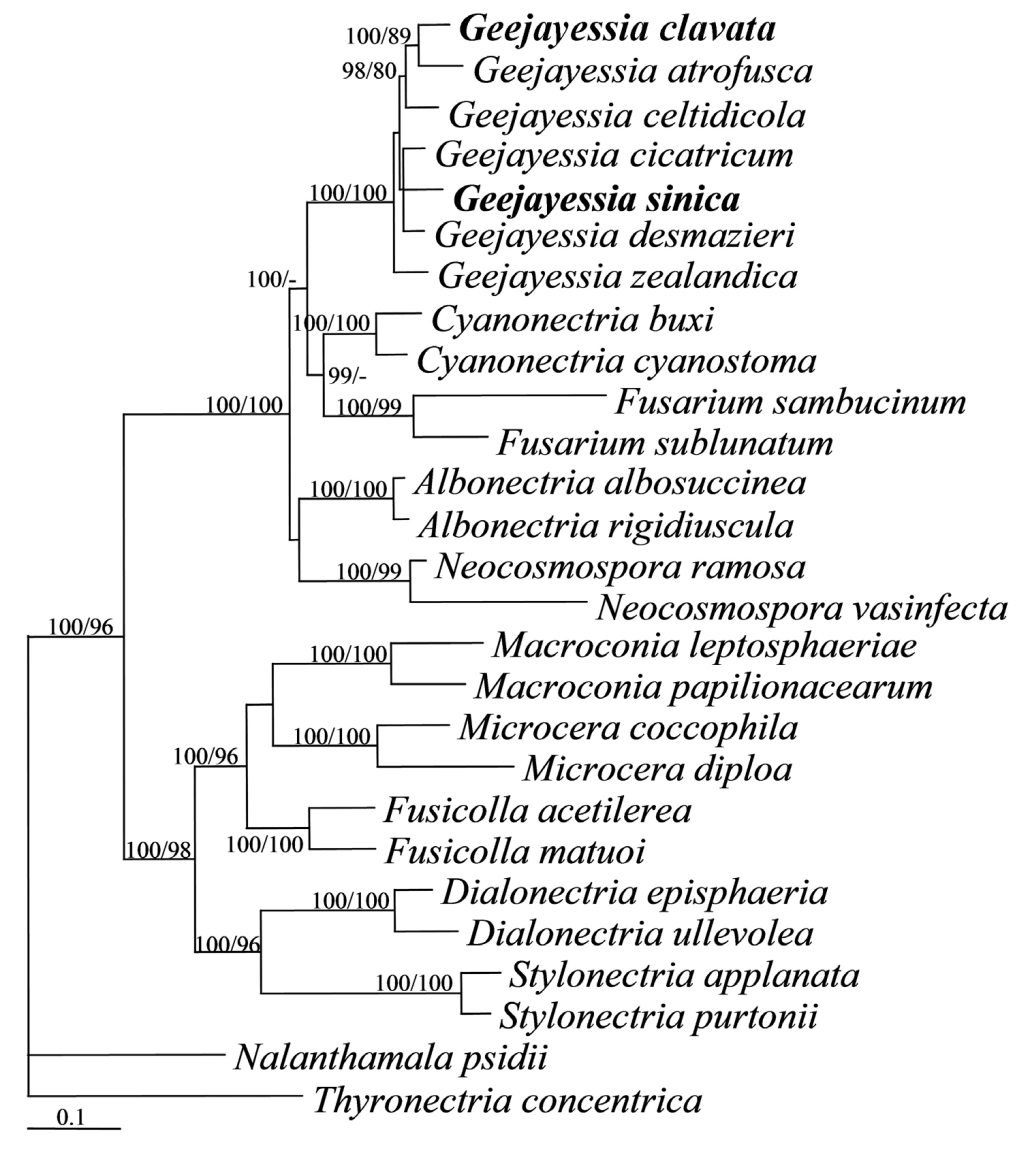
A Bayesian Inference trees inferred from the combined ACL1, ITS and RPB2 sequences. BIPP (left) above 90% and MPBP (right) above 50% are indicated at nodes.

The ACL1, ITS and RPB2 sequences of 25 taxa belonging to 10 genera having fusarium-like asexual states were analysed through the methods of BI and MP. The PHT (P = 0.01) indicated that the individual partitions were not highly incongruent (Cunningham 1997); the three loci were thus combined for phylogenetic analyses. The combined datasets include 2258 characters, of which 1085 were constant, 173 variable and parsimony-uninformative and 1000 parsimony-informative. The MP analysis resulted in a single most parsimonious tree (tree length = 4885, CI = 0.4491, HI = 0.5509, RI = 0.5638, RCI = 0.2532). The final matrix was deposited in TreeBASE with accession No. S20853. The BI tree generated is shown (Figure 1). The topology of the BI tree is similar to that of the MP tree. The 25 investigated species were grouped together (BIPP/MPBP = 100%/96%) and further segregated into two main clades (Figure 1). Species of *Geejayessia* clustered into one clade together with *Albonectria* Rossman & Samuels, *Cyanonectria* Samuels & P. Chaverri, *Fusarium* and *Neocosmospora* E.F. Sm. (BIPP/MPBP = 100%/100%) and those of *Dialonectria* (Sacc.) Cooke, *Fusicolla* Bonord., *Macroconia* (Wollenw.) Gräfenhan, Seifert & Schroers, *Microcera* Desm. and *Stylonectria* Höhn. formed another clade (BIPP/MPBP = 100%/98%). HMAS 248725, HMAS 248726 and other representatives of *Geejayessia* formed a highly supported monophyletic group (BIPP/MPBP = 100%/100%), which confirmed their taxonomic positions in the genus.

### Taxonomy

#### Geejayessia clavata

Taxon classificationFungiHypocrealesNectriaceae

Z.Q. Zeng & W.Y. Zhuang sp. nov.

Figures 2 , 3

##### Holotype.

CHINA, Henan Province, Longyuwan, 33°40'45"N, 111°46'26"E, alt. 1500 m, on bark of *Buxus* sp., 17 September 2013, H.D. Zheng, Z.Q. Zeng & Z.X. Zhu 8728 (holotype: HMAS 275654), dried ex-type culture HMAS 248725.

##### Etymology.

The specific epithet refers to the clavate microconidia.

##### Description.

Mycelium not visible around ascomata or on host. Ascomata perithecial, crowded in group of 5 to 40, on basal stroma, oval, subglobose to globose, smooth, bright red when fresh, red brownish to dark red when dry, with a darker red ostiolar region, turning purple red in KOH and orange yellow in LA, 128–175 × 206–255 μm (n = 17). Perithecial wall consisting of a single layer, 15–25 μm thick, cells forming textura prismatica, 2–12 × 2–6 μm, walls 1–1.2 μm thick. Asci cylindrical, with a rounded apex, (4–7-)8-spored, 55–75 × 5–9 μm. Ascospores ellipsoidal to broadly ellipsoidal, equally 2-celled, slightly constricted at septum, smooth or finely verruculose, hyaline or pale brown, obliquely uniseriate in ascus often with ends overlapping, 7.5–12 × 4.5–5.5 μm.

##### Culture characteristics.

Colony on PDA 48 mm diam. after 7 d at 25 °C, surface cottony, aerial mycelium white, producing vinaceous pigment in medium. Colony on SNA 30 mm diam. after 7 d at 25 °C, surface slightly floccose, with sparse whitish aerial mycelium. Colony on CMD 56 mm diam. after 7 d at 25 °C, surface floccose, with sparse whitish aerial mycelium, producing vinaceous pigment in medium. Conidiophores with short simple branches. Conidiogenous cells monophialidic, cylindrical, tapering toward the tip, 12–63 × 1.5–3.5 μm. Conidia clavate, not in chains, hyaline, aseptate, 4–7 × 0.8–2 μm (n = 60). Macroconidia and chlamydospores not observed.

**Notes.** Attempts were made to obtain macroconidia of the fungus in culture, but failed. Although the falcate macroconidia are lacking, the major phenotypic features of the fungus, such as occurrence on bark of *Buxus* sp., perithecia broadly ampulliform with a short neck, asci cylindrical with a rounded apex, ellipsoidal ascospores uniseptate and conidiophores monophialidic, fit well with the generic concept of *Geejayessia*. The molecular data confirm the taxonomic placement and indicate its close relationship with *G.atrofusca* (Figure 1, BIPP/MPBP = 100%/89%). *Geejayessiaatrofusca* differs significantly in dark brown to black ascomata that do not change colour in KOH or LA, wider asci [(7.5-)9.8-13.3(-15) μm wide] and longer ascospores [(10-)11.2-14.2(-17.0) μm long]. Its microconidia are oblong to slightly curved and falcate but not clavate and are longer and wider (Samuels and Rogerson 1984).

**Figure 2.**
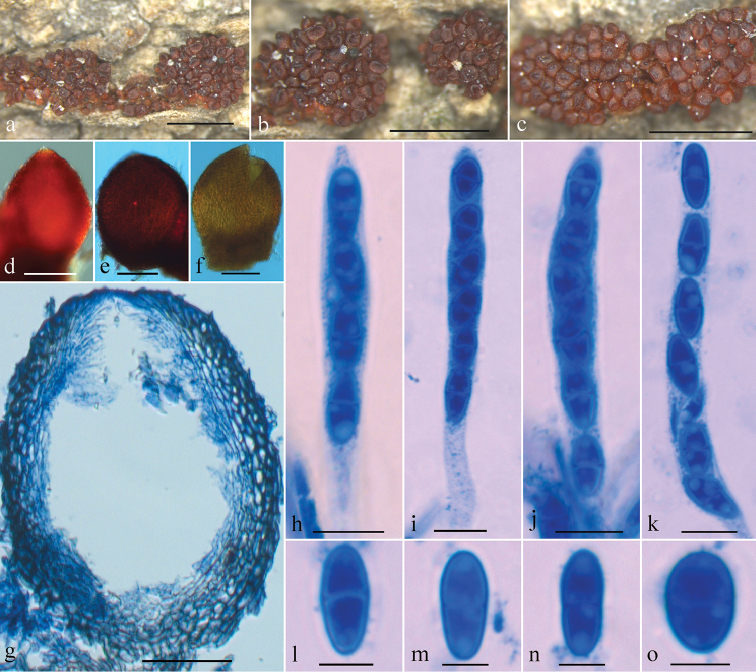
*Geejayessiaclavata* sexual state (holotype, HMAS 275654): **a–c** Ascomata on natural substrate **d–f** colour of perithecium in water (**d**), 3% KOH (**e**) and 100% lactic acid (**f**) **g** median section through perithecium **h–k** Asci with ascospores **l–o** Ascospores. Scale bars: 1 mm (**a–c**); 100 μm (**d–f**); 50 μm (**g**); 10 μm (**h–k**), 5 μm (**l–o**).

**Figure 3.**
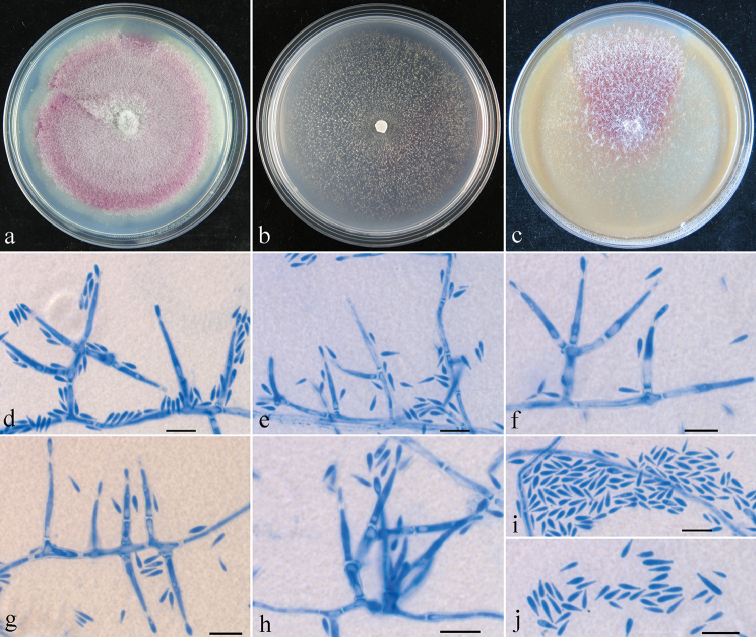
*Geejayessiaclavata* asexual state (HMAS 248725): **a–c** colony on PDA (**a**) SNA (**b**) and CMD (**c**) **d–j** conidiophores, phialides and/or microconidia on SNA. Scale bar: 10 μm (**d–j**).

#### Geejayessia sinica

Taxon classificationFungiHypocrealesNectriaceae

Z.Q. Zeng & W.Y. Zhuang sp. nov.

Figures 4 , 5

##### Type.

CHINA, Hubei Province, Shennongjia, 31°29'17"N, 110°20'58"E, alt. 2800 m, on bark of *Buxus* sp., 15 September 2014, Z.Q. Zeng, H.D. Zheng, W.T. Qin & K. Chen 9606 (holotype: HMAS 254520), dried ex-type culture HMAS 248726.

##### Etymology.

Specific epithet refers to the type locality China.

##### Description.

Mycelium not visible around ascomata or on host. Ascomata perithecial, solitary or in groups of 5 to 40, with a basal stroma, pyriform or subglobose to globose, smooth, collapsing laterally when dry, red to bright red with a dark red ostiolar region, turning dark purple red in KOH and light yellow in LA, 255–343 × 176–314 μm (n = 14). Perithecial wall of a single layer, 18–38 μm thick, of textura prismatica, cells 8–23 × 2–6 μm, walls 1.2–1.5 μm thick. Asci subcylindrical to clavate, with a rounded apex, 6(–8)-spored, 88–123 × 7–10(–12.5) μm. Ascospores ellipsoidal, hyaline or pale brown, smooth or finely warted, bicellular, slightly constricted at septum, obliquely uniseriate, 10–18(–20) × 5–7.5 μm.

##### Culture characteristics.

Colony on PDA 42 mm diam. after 7 d at 25 °C, surface cottony, with whitish aerial mycelium, forming concentric rings, with pale vinaceous pigment produced in medium. Colony on SNA 26 mm diam. after 7 d at 25 °C, surface slightly velvet, with sparse whitish aerial mycelium. Colony on CMD 40 mm diam. after 7 d at 25 °C, surface radial, slightly floccose, with sparse whitish aerial mycelium. Conidiophores with short simple branches. Conidiogenous cells monophialidic, cylindrical, slightly tapering toward the tip, indefinite in length. Macroconidia falcate, with an arcuate tip and a pedicellate foot cell, hyaline, (3–4–)5-septate, 3-septate: 30–53 × 4–5 μm, 4-septate: 50–60 × 4.5–5.2 μm, 5-septate: 53–80 × 4.6–5.3 μm. Microconidia and chlamydospores not observed.

##### Notes.

*Geejayessiasinica* is phylogenetically related to and morphologically similar to *G.cicatricum* and *G.desmazieri* in perithecial gross morphology, subcylindrical to clavate asci, ellipsoidal to broadly ellipsoidal, uniseptate ascospores, falcate macroconidia (Schroers et al. 2011). *Geejayessiacicatricum* differs from *G.sinica* in having smaller perithecia (160–260 × 125–250 μm) and ascospores [(9.5–)11.5–13(–14.5) × (4.5–)5.0–6(–6.5) μm], thinner perithecial wall [(12–)13.5–18(–21) μm thick), shorter asci [(65.5–)73–92.5(–103) μm long), macroconidia with more septa [(2–)5–7(–8)] and slow growth on PDA (15–20 mm diam. after 7 d at 25 °C) (Schroers et al. 2011). *Geejayessiadesmazieri* is distinguished by shorter asci [(75.5–)85(–100) μm long], smaller ascospores [(9.5–)11–12.5(–15) × (4.5–)5.5–6(–7) μm] and slow growth on PDA (20 mm diam. after 7 d at 25 °C) (Schroers et al. 2011). The ITS sequence of *G.sinica* differs from that of the other two species by 29 bp and 29 bp divergences in total length of 521 bp. The protein-encoding gene sequences of *G.sinica* differ from those of *G.cicatricum* (*G.desmazieri*) by 59 (66) bp differences of 815 bp long ACL1 fragment and 34 (35) bp differences of the 672 bp long RPB2 region.

**Figure 4.**
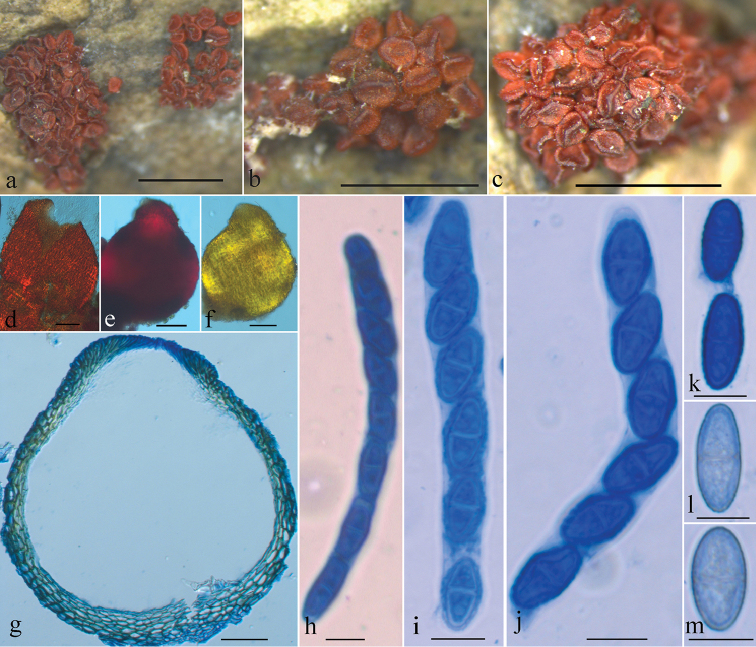
*Geejayessiasinica* sexual state (holotype, HMAS 254520): **a–c** ascomata on natural substrate **d–f** colour of perithecium in water (**d**), 3% KOH (**e**) and 100% lactic acid (**f**) **g** median section through perithecium **h–j** asci with ascospores **k–m** ascospores. Scale bars: 1 mm (**a–c**); 100 μm (**d–f**); 50 μm (**g**); 10 μm (**h–m**).

**Figure 5.**
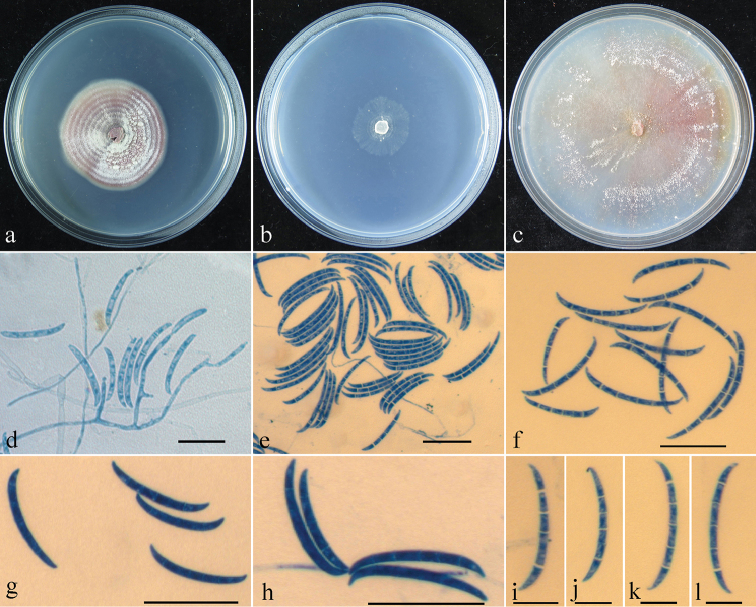
*Geejayessiasinica* asexual state (HMAS 248726): **a–c** colony on PDA (**a**), SNA (**b**) and CMD (**c**) **d** conidiophores, conidiogenous cells and macroconidia on SNA**e–l** Macroconidia on SNA. Scale bars: 50 μm (**d–h**); 10 μm (**i–l**).

## Discussion

Schroers et al. (2011) recognised five species of *Geejayessia*. *Geejayessiamontana* Lechat & J. Fourn was recently described and its placement was supported by morphological characteristics of both sexual and asexual states, as well as analysis of ITS sequences (Lechat and Fournier 2017). Meanwhile, a new combination, *G.hispanica* (Lechat & Priou) Lechat & J. Fourn was proposed based on the ITS sequence of *Geejayessia* sp. BRFM 1015 (GenBank accession no. JX082350) (Lechat and Fournier 2017). However, ‘*Geejayessiahispanica*’ grows on *Phoenixcanariensis* rather than *Buxus*, *Celtis* or *Staphylea*, which deviates from the original generic concept of the genus (Schroers et al. 2011). This fungus was treated as *Cosmosporahispanica* Lechat & Priou in the present study. *Cosmosporamatuoi* Hosoya & Tubaki was also combined with *Geejayessia* as *G.matuoi* (Hosoya & Tubaki) Lechat & Rossman (Lechat and Rossman 2017). Nevertheless, Gräfenhan et al. (2011) and Lombard et al. (2015) treated *Cosmosporamatuoi* as a member of *Fusicolla*, which is followed in this study. To clarify the taxonomic positions of ‘*G.hispanica*’ and ‘*G.matuoi*’, more evidence is certainly required.

According to the International Code of Nomenclature for algae, fungi and plants (McNeill et al. 2012), the name *Fusarium* is accepted as the correct generic name for fungi with *Gibberella* Sacc. sexual states (Rossman et al. 2013). The asexual states of other genera are marked as fusarium-like (Lombard et al. 2015). In the present study, the phylogeny, based on analyses of the combined ACL1, ITS and RPB2 sequences, recognised nine clades amongst the investigated taxa which are in accordance with the genera *Albonectria*, *Cyanonectria*, *Dialonectria*, *Fusicolla*, *Geejayessia*, *Macroconia*, *Microcera*, *Neocosmospora* and *Stylonectria*. This result is basically consistent with that by Schroers et al. (2011).

Joining the two new species to the *Geejayessia* clade, the tree topology (Figure 1) remains basically the same as that revealed by Schroers et al. (2011). Our result showed *G.clavata* and *G.atrofusca* both forming microconidia in culture, grouped together with relatively high statistical supports (Figure 1, BIPP/MPBP = 100%/89%). *Geejayessiasinica*, *G.cicatricum* and *G.desmazieri*, as sister-groups, are poorly supported (BIPP/MPBP less than 50%).

Host specificity has been shown in some fungi of Nectriaceae; for example, *Thyronectriaaurigera* (Berk. & Ravenel) Jaklitsch & Voglmayr occurs only on Oleaceae, *T.berolinensis* (Sacc.) Seaver on *Ribes* and *T.aquifolii* (Fr.) Jaklitsch & Voglmayr on *Ilexaquifolium* (Jaklitsch and Voglmayr 2014; Zeng and Zhuang 2016). Species of *Geejayessia* are also host-specific. As known currently, *G.clavata*, *G.sinica*, *G.cicatricum* and *G.desmazieri* occur only on *Buxus* spp., *G.celtidicola* only on *Celtisoccidentalis* and *G.atrofusca* only on *Staphyleatrifolia* (Schroers et al. 2011).

The genus *Geejayessia* was previously known from Europe, North America and Oceania (Samuels and Rogerson 1984; Nirenberg and Samuels 2000; Schroers et al. 2011). The new species discovered from central China extends the distribution of the genus to Asia.

## Supplementary Material

XML Treatment for Geejayessia clavata

XML Treatment for Geejayessia sinica
